# Tooth Implantation Following Socket Preservation Using Diode Laser Treatment Combined With “Hood” Closure Technique: A Case Report

**DOI:** 10.1155/crid/1066299

**Published:** 2026-06-24

**Authors:** Shanshan Ma, Kui Su, Rongyu Huang, Xue Gao, Yan Wang

**Affiliations:** ^1^ Department of Periodontology, Hospital of Stomatology, Zhongshan City, Zhongshan, Guangdong, China; ^2^ Department of Oral Mucosa, Hospital of Stomatology, Zhongshan City, Zhongshan, Guangdong, China; ^3^ Department of Orthodontics, Jixi Stomatological Hospital, Jixi, Heilongjiang, China; ^4^ Department of Oral and Maxillofacial Surgery, Sun Yat-sen Memorial Hospital, Sun Yat-sen University, Guangzhou, Guangdong, China, sysu.edu.cn

## Abstract

**Objective:**

The aim of this study is to evaluate the efficacy of diode laser‐assisted socket preservation with “hood” closure for dental implantation in advanced periodontitis.

**Methods:**

A 31‐year‐old female with Stage III Grade C periodontitis underwent minimally invasive extraction of Tooth #46, followed by combined laser therapy and flap closure for socket preservation.

**Results:**

At 2‐year follow‐up, the results demonstrated: (1) complete resolution of inflammation (BOP 0%); (2) Elimination of pathological pockets (PD ≥ 4 mm 0%); and (3) successful implant placement with stable bone volume.

**Conclusion:**

This technique ensures optimal wound healing and ridge preservation, enhancing implant outcomes in compromised sites.

## 1. Introduction

Socket preservation refers to protective or restorative interventions performed during or after tooth extraction to minimize alveolar bone resorption, preserve soft and hard tissue, thus providing a reliable foundation for future dental prosthetic restoration [1]. However, conventional socket preservation techniques often face challenges in achieving primary wound closure. This may lead to complications such as graft exposure, graft material loss, and potential secondary infection [2].

The “hood” closure technique involves elevating a full‐thickness mucoperiosteal flap composed of granulation tissue along with its overlying soft tissue from the alveolar bone surface, which is then mobilized to the gingival margin for socket coverage [3]. This technique ensures reliable wound healing. Yet the granulation tissue flap may lead to prolonged postoperative bleeding and significant pain.

Diode laser demonstrates significant bactericidal effects against obligate anaerobes, reduces inflammatory response, and enhances periodontal tissue attachment. Clinically, it can markedly decrease postoperative bleeding and pain following flap surgery.

This report describes the treatment of a 31‐year‐old female patient with advanced chronic periodontitis (Stage III, Grade C), focusing on the application of a diode laser‐assisted socket preservation technique with “hood” closure to address severe alveolar bone loss and facilitate successful dental implantation. The case illustrates the clinical challenges in managing periodontally compromised sites and the efficacy of this minimally invasive approach in achieving stable wound healing, ridge preservation, and long‐term implant success, as detailed in the subsequent case presentation.

## 2. Case Report

### 2.1. Patient

In July 2022, a 31‐year‐old female was referred to the Department of Periodontology, Hospital of Stomatology, Zhongshan City, China, with chief complaint of recurrent gingival swelling and pain in the right posterior mandible for several months. The patient reported gingival swelling, pain, and tooth mobility in the right posterior mandible for 3 months, accompanied by masticatory weakness. Previous scaling at a local dental clinic provided no improvement in mobility. Recent recurrence of swelling and pain prompted this visit. The patient had no significant systemic diseases or drug allergies.

### 2.2. Clinical Examination

The clinical examination of this study is described as follows:

• Oral hygiene status: poor.
• Gingival condition: dark red, swollen, thickened, and spongy texture.
• Bleeding on Probing (BOP): positive.
• Pigmentation (plague staining): ++.
• Soft deposits: Index II° (moderate accumulation).
• Supragingival calculus: Index I° (mild).
• Subgingival findings: Abundant subgingival calculus and periodontal pockets detected.
• Tooth mobility:
  o. Grade I: #16, #25.
  o. Grade II: #17, #26, #27.
  o. Grade III: #46 (with purulent exudate from deep pockets).

### 2.3. Initial Periodontal Charting

The initial periodontal charting of this study is presented as follows:

• Bleeding sites: 42%.
• Pocket depth (PD) ≥ 4‐mm sites: 17% (Figure 1).

**Figure 1:**
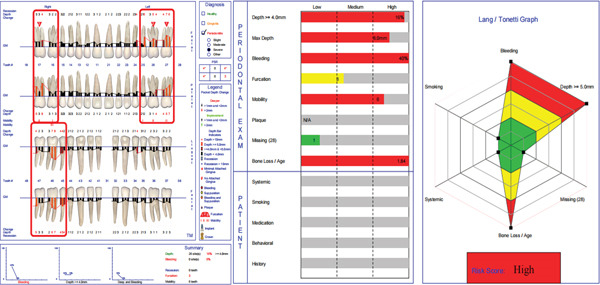
Initial periodontal charting. Initial periodontal charting reveals 42% bleeding sites (BOP) and 17% deep pockets (PD ≥ 4 mm), indicating severe periodontal inflammation and attachment loss at Site #46 (mandibular right first molar, red‐highlighted), necessitating socket preservation.

### 2.4. Auxiliary Examination

#### 2.4.1. CBCT Findings

The CBCT findings of this study are presented as follows:

• Generalized alveolar bone loss: Crestal height reduction observed throughout the dentition.
• Horizontal bone resorption: Ranging from 1/3 to 2/3 of the root length.
• Severe bone loss (tooth‐specific): #17, #26, and #46: Bone resorption extending to the periapical region (indicating advanced periodontal destruction) (Figures 2 and 3).

**Figure 2:**
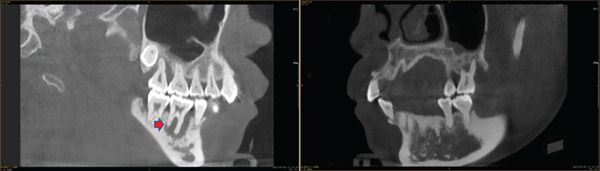
CBCT findings. CBCT scans reveal generalized alveolar bone loss (crestal height reduction), horizontal resorption (1/3–2/3 root length), and severe periapical bone loss at Teeth #17, #26, and #46 (red‐marked), confirming advanced periodontal destruction that necessitated socket preservation at #46.

**Figure 3:**
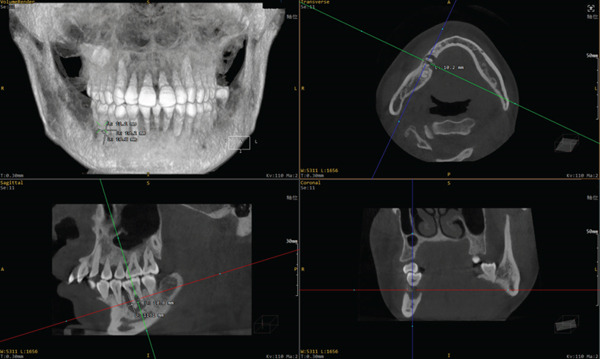
Preoperative measurement of defect area of #46.

### 2.5. Diagnosis

#### 2.5.1. Periodontitis (Stage III, Grade C)

##### 2.5.1.1. Treatment Plan

The treatment plan of this study is presented as follows:

1. Initial therapy phase:
  • Professional oral hygiene instruction (OHI).
  • Full‐mouth scaling and root planing (SRP).
  • Laser‐assisted periodontal pocket disinfection.
2. Surgical phase:
  • Flap surgery with bone grafting for Teeth #16–17 and #25–27.
  • Minimally invasive extraction of #46 with simultaneous ridge preservation (socket preservation).
  • The bone substitute material used in this case is Geistlich Bio‐Oss, a porous bovine bone mineral with a particle size of 0.25–1.0 mm (0.5 g≈1 cm^3^). Concentrated growth factors (CGF) were prepared from the patient′s venous blood and mixed with Geistlich Bio‐Oss particles to form a viscous and moldable bone graft composite. The composite was gently compacted and implanted into the extraction socket, and a CGF membrane was then placed on the surface of the graft area to promote primary wound closure and bone regeneration.
3. Implant rehabilitation phase:
  • Implant placement at #46 site after periodontal stabilization and ridge healing (Figure 4).
4. Supportive periodontal therapy (SPT):
  • Regular follow‐up visits (3–4 months) for long‐term maintenance (Figures 5, 6, 7, 8, and 9).

**Figure 4:**
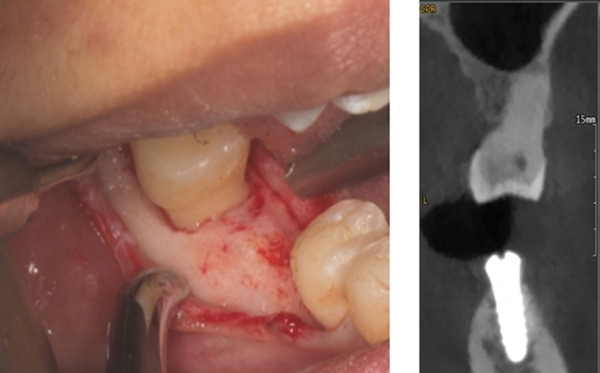
Twelve‐month postimplantation at Site #46. Intraoral view shows stable implant restoration with healthy soft tissue. CBCT confirms osseointegration (no peri‐implant bone loss), validating long‐term success.

**Figure 5:**
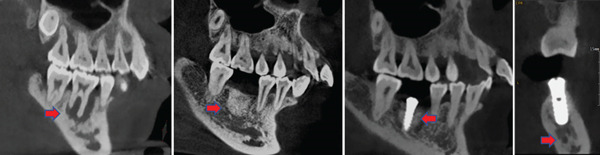
CBCT comparison shows severe pretreatment bone loss versus improved bone volume postsocket preservation, validating technique efficacy.

**Figure 6:**
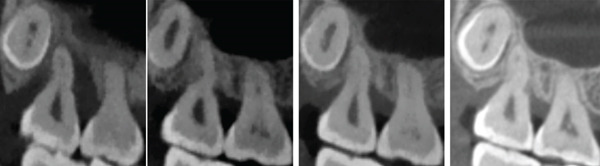
CBCT series at baseline, 6 months, 1 year, and 2 years postoperation show progressive bone regeneration around #17, demonstrating long‐term efficacy of flap bone grafting.

**Figure 7:**
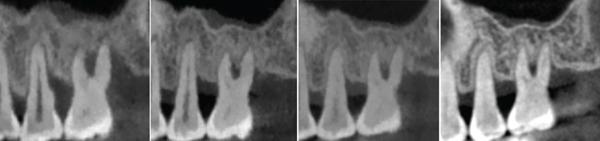
CBCT images over 2 years reveal bone remodeling and stable regeneration at #25–26 following flap bone grafting, confirming sustained treatment outcomes.

**Figure 8:**
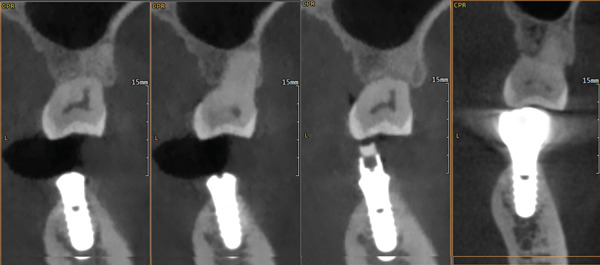
Twelve‐month CBCT after socket preservation shows successful osseointegration and stable crown restoration at #46, validating implant procedure efficacy.

**Figure 9:**
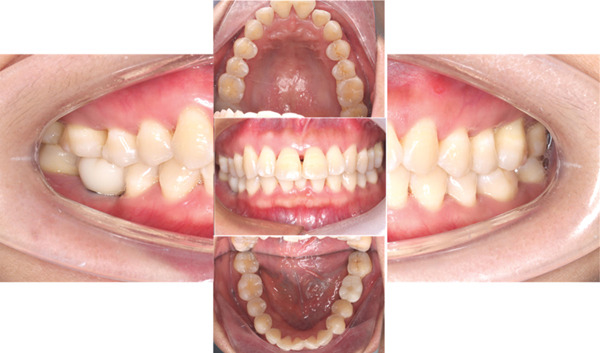
Intraoral images show healthy gingiva and well‐integrated restorations at completion, demonstrating successful periodontal control and implant esthetics.

##### 2.5.1.2. Two‐Year Posttreatment Periodontal Examination Summary

The 2‐year posttreatment periodontal examination summary is described as follows:

• BOP: 42% → 0% (full resolution of inflammation).
• PD ≥4‐mm sites: 17% → 0% (elimination of pathological pockets).
• Risk assessment: “High” → “Low” (controlled disease activity) (Figure 10).

**Figure 10:**
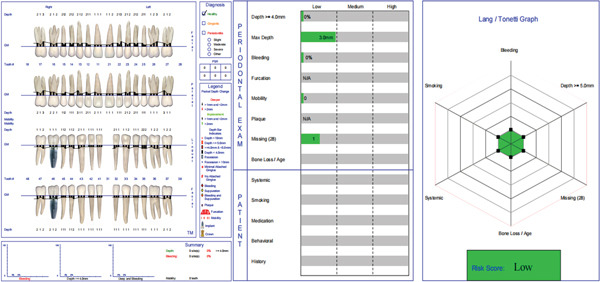
Two‐year Posttreatment periodontal assessment. Two years after treatment, periodontal charts show BOP dropped from 42% to 0%, PD ≥4‐mm sites from 17% to 0%. Risk assessment shifted from “High” to “Low”, proving treatment′s long‐term efficacy.

## 3. Discussion

Ridge preservation refers to procedures performed during or immediately after tooth extraction to minimize alveolar ridge resorption, thereby maintaining sufficient bone volume and optimal bone quality for future implant placement. [1]. Common clinical approaches include:

• Selecting appropriate extraction timing.
• Minimally traumatic extraction techniques (to reduce alveolar bone damage).
• Immediate socket grafting with bone or bone substitute materials.
• Primary soft tissue closure [2].

Among these, socket grafting using bone grafts or bone substitutes has become the most widely adopted clinical method due to its technical simplicity, low morbidity, and material availability. Current protocols typically combine atraumatic extraction with bioactive materials to enhance bone and soft tissue regeneration at the extraction site. This approach effectively preserves the ridge contour of both hard and soft tissues, creating favorable conditions for subsequent implant rehabilitation [3].

Adequate wound closure is a critical factor for successful ridge preservation [4]. Current socket sealing techniques primarily include:

### 3.1. Barrier Membranes

The application of guided bone regeneration (GBR) principles in ridge preservation requires membrane placement to

• Protect the blood clot/graft material from epithelial/connective tissue invasion.
• Prevent microbial contamination of the socket.
• Maintain space for new bone formation [5].

Clinical membrane classifications are as follows:

• Resorbable membranes (e.g., collagen and polyglycolic acid).
• Nonresorbable membranes (e.g., ePTFE and titanium‐reinforced) (*Bio-Gide collagen membrane is widely used clinically*).

### 3.2. Autogenous Soft Tissue Management

Early protocols emphasized primary closure for

• Membrane protection (if used).
• Infection control.
• Optimal wound healing.

Surgical techniques are as follows:

• Coronally advanced flap.
• Pedicle flaps.
• Free gingival grafts.
• Connective tissue grafts [6].

The latter three methods not only achieve primary closure but also increase keratinized tissue width and thickness.

Although barrier membrane placement and primary wound closure are widely used in ridge preservation, several clinical challenges persist:

1. Altered mucogingival junction: Coronally advanced flaps for tension‐free closure displace the mucogingival junction, potentially compromising prosthetic emergence profiles and esthetic outcomes.
2. Autogenous tissue graft drawbacks are as follows:
  • Technical sensitivity: requires advanced surgical skills.
  • Secondary surgical site morbidity (e.g., palatal harvesting).
  • Graft failure risks: Necrosis may lead to fibrous healing, increasing patient discomfort and treatment costs.
3. Early healing complications are presented as follows:
  • Flap elevation and coronal advancement to achieve primary intention healing often result in:
    • Progressive keratinized tissue loss.
    • Wound dehiscence (under excessive tension).
    • Membrane exposure (↑ risk of infection/graft failure) [7].

This study introduces an innovative “hood” closure technique, where reactive soft tissue within the extraction socket is utilized for wound closure following minimally invasive tooth extraction. The “hood” closure technique is a minimally invasive surgical technique used in extraction socket management and implant‐related surgery, aiming to achieve tension‐free primary wound closure. The technique involves preparing a partial‐thickness or full‐thickness mucoperiosteal flap, advancing and suturing it to form a protective “hood‐like” coverage over the entrance of the extraction socket without extensive flap elevation or extensive relaxation of the surrounding tissues.

### 3.3. Comparison With Traditional Techniques

Traditional extraction socket closure techniques often rely on extensive elevation of the mucoperiosteal flap, which is prone to tissue trauma, postoperative pain, gingival recession, and impaired blood supply, thus affecting soft tissue healing and alveolar ridge preservation. In contrast, the “hood” closure technique can reduce the range of flap elevation, maintain local blood supply, stabilize the wound environment, and is especially suitable for complex sites with poor periodontal conditions, thin gingival biotype, or anatomical limitations.

The advantages are listed as follows:

• Achieves primary intention healing without extensive flap mobilization.
• Preserves existing keratinized tissue by avoiding coronal flap advancement.

The disadvantages are described as follows:

• Potential complications associated with granulation tissue:
  ▪ Persistent postoperative hemorrhage.
  ▪ Significant pain (compared with conventional closure methods).
  ▪ Limited predictability in wide/sclerotic sockets.

This study combined the “hood” closure technique with diode laser‐assisted disinfection and bone augmentation with bone graft material combined with CGF, which further optimized the wound closure effect, reduced the risk of infection and wound dehiscence, improved the soft tissue morphology, and achieved predictable alveolar ridge preservation.

The diode laser is a solid‐state semiconductor laser that typically combines gallium, arsenide, and other elements (e.g., aluminum and indium) to convert electrical energy into light energy [8].

Key advantages in dental applications (Table 1) are presented as follows:

1. Minimal interaction with tooth structure:
  • Does not affect dental hard tissues, making it ideal for soft tissue surgery.
  • More cost‐effective compared with other laser systems.
2. Superior hemostasis and tissue interaction:
  • Exhibits greater absorption and less penetration than Nd:YAG lasers, particularly in blood‐rich tissues, reducing collateral damage [9].
  • Wavelength is absorbed by hemoglobin, leading to:
    • Tissue coagulation.
    • Formation of a carbonized layer (aiding in hemostasis).
  • Thermal coagulation of blood vessels enhances its hemostatic efficiency.
3. Clinical benefits are listed as follows:
  • Reduced postoperative bleeding and swelling.
  • Precise soft tissue cutting with minimal thermal damage.
  • Faster healing due to controlled coagulation.

**Table 1:** Comparison of diode laser, ND:YAG laser, and CO₂ laser in dental applications.

Feature	Diode laser	ND:YAG laser	CO_2_ laser
**Penetration depth**	Low	High	Moderate
**Hemostasis**	Excellent	Moderate	Poor
**Hard tissue interaction**	None	Possible	High
**Cost efficiency**	High	Moderate	Low

In this case, the specific parameters and operation protocol of the diode laser are as follows: a 980 nm diode laser was used to disinfect the extraction socket in the subgingival pocket disinfection (SP) mode with an output power of 1.7 W. Each site was irradiated for 30 s, with 1–3 treatment sessions in total.

## 4. Conclusion

Due to its tissue coagulation and hemostatic properties, the diode laser is an excellent choice for soft tissue procedures in dentistry, offering precision, safety, and cost‐effectiveness.

### 4.1. Diode Laser‐Assisted “Hood” Closure Technique for Ridge Preservation

The combination of diode laser and the “hood” closure technique for ridge preservation achieves primary wound healing while avoiding complications such as persistent postoperative hemorrhage. Furthermore, it promotes optimal hard tissue regeneration, establishing a solid foundation for subsequent implant placement and significantly enhancing the long‐term outcomes of implant‐supported prostheses.

Key advantages of this combined approach are presented as follows:

1. Enhanced hemostasis and soft tissue management
  • The diode laser ensures effective coagulation, minimizing bleeding risks associated with the reactive granulation tissue in the socket.
  • The “hood closure” technique preserves keratinized mucosa without extensive flap mobilization.
2. Superior bone healing and ridge preservation
  • Reduces postoperative inflammation, facilitating undisturbed bone remodeling.
  • Maintains socket architecture, preventing alveolar ridge collapse.
3. Improved implant success and prosthetic outcomes
  • Provides adequate bone volume for stable implant placement.
  • Ensures favorable soft tissue contours for esthetic and functional prosthetic rehabilitation.

### 4.2. Clinical Significance

This minimally invasive protocol synergizes the benefits of:

• Diode laser (precise soft tissue incision, coagulation, reduced trauma).
• “Hood” closure technique (preservation of keratinized tissue, primary closure).

Within the limitations of this case report, the combined application of diode laser‐assisted debridement, socket preservation with Bio‐Oss and CGF, and the hood closure technique achieved favorable clinical outcomes. The treatment achieved complete soft tissue healing, effective alveolar ridge preservation, and successful osseointegration of the subsequent implant. This minimally invasive treatment protocol has good predictability and can provide a valuable clinical option for preimplant restoration of extraction sockets with poor periodontal conditions.

## Author Contributions

Shanshan Ma contributed to the data collection, manuscript writing, and overall study coordination. Kui Su participated in treatment planning and periodontal management. Rongyu Huang assisted with surgical procedures and case follow‐up. Xue Gao provided administrative support and participated in data organization. Yan Wang designed the research, supervised the study, and provided critical revisions to the manuscript.

## Funding

No funding was received for this manuscript.

## Ethics Statement

This study was approved by the Medical Ethics Committee of the Hospital of Stomatology, Zhongshan City (Ethics Approval No.:2024‐14). Written informed consent was obtained from the patient for the publication of this case report and accompanying images.

## Consent

The manuscript is approved by all authors for publication.

## Conflicts of Interest

The authors declare no conflicts of interest.

## Data Availability

The data that support the findings of this study are available from the corresponding author upon reasonable request. The data are not publicly available due to privacy or ethical restrictions.
